# Effects of sugarcane aphid herbivory on transcriptional responses of resistant and susceptible sorghum

**DOI:** 10.1186/s12864-018-5095-x

**Published:** 2018-10-26

**Authors:** Mahnaz Kiani, Adrianna Szczepaniec

**Affiliations:** Department of Entomology, Texas A&M AgriLife Research, 6500 Amarillo Blvd. W, Amarillo, TX 79106 USA

**Keywords:** *Melanaphis sacchari*, *Sorghum bicolor*, Plant-insect interaction, RNA-seq, Plant defenses

## Abstract

**Background:**

Sugarcane aphid (*Melanaphis sacchari*) outbreaks in sorghum that were first reported in 2013 are now the most significant threat to this crop in all major sorghum production areas in the U.S. The outcomes of interactions between sugarcane aphid and sorghum and thus the severity of the outbreaks depend on sorghum genotype and potentially also on the phenology of sorghum. Mechanisms underlying these interactions are not known, however. Thus, the goal of this research was to characterize transcriptional changes in a commercially available resistant and a susceptible genotype of sorghum at 2- and 6-wk post-emergence exposed to *M. sacchari* herbivory. The effects of sorghum age and genotype on the daily change in aphid densities were also evaluated in separate greenhouse experiments.

**Results:**

A higher number of diffentially expressed genes (DEGs) was recovered from the 2-wk plants exposed to aphid herbivory compared to the 6-wk plants across genotypes. Further, gene ontology and pathway analysis indicated a suite of transcriptional changes in the resistant genotype that were weak or absent in the susceptible sorghum. Specifically, the aphid-resistant genotype exposed to *M. sacchari* up-regulated several genes involved in defense, which was particularly evident in the 2-wk plants that showed the most robust transcriptional responses. These transcriptional changes in the younger resistant sorghum were characterized by induction of hormone-signaling pathways, pathways coding for secondary metabolites, glutathion metabolism, and plant-pathogen interaction. Furthermore, the 2-wk resistant plants appeared to compensate for the effects of oxidative stress induced by sugarcane aphid herbivory with elevated expression of genes involved in detoxification. These transcriptional responses were reflected in the aphid population growth, which was significantly faster in the susceptible and older sorghum than in the resistant and younger plants.

**Conclusion:**

This experiment provided the first insights into molecular mechanisms underlying lower population growth of *M. sacchari* on the resistant sorghum genotype. Further, it appears that the younger resistant sorghum was able to mount a robust defense response following aphid herbivory, which was much weaker in the older sorghum. Several pathways and specific genes provide specific clues into the mechanisms underlying host plant resistance to this invasive insect.

**Electronic supplementary material:**

The online version of this article (10.1186/s12864-018-5095-x) contains supplementary material, which is available to authorized users.

## Background

Plants have evolved diverse strategies to defend, tolerate, or avoid insect herbivores [1, 2] that provide valuable resources for deploying in pest management [3, 4]. When plants detect the injury inflicted by herbivores, they fine-tune their responses through a flexible and finely balanced network that in turn activates specific defense responses [4, 5] with a large intraspecific variation in signaling cascades and production of metabolites [4]. The genetic diversity of herbivore-resistant traits is also a valuable resource for studying the mechanism of resistance and the ultimate deployment of them in crop breeding programs [4, 6]. In fact, resistant cultivars and hybrids have been employed as an effective method for controlling economically damaging arthropods in many different crops, including sorghum [*Sorghum bicolor* (L.) Moench] [7–11], which is vulnerable to significant injury caused by diverse insect pests [12]. The employment of host-plant resistance in controlling greenbug, *Schizaphis graminum* Rodandi (Hemiptera: Aphididae)*,* in sorghum is an example of an effective integrated pest management in this crop [13–16]. As a result of the selected resistance sources that were bred into commercial sorghum hybrids, the frequency of chemical control to suppress the pest was significantly reduced [17, 18].

Invasive pests pose unique challenges to crop protection, which was recently exemplified by an invasion of sugarcane aphid, *Melanaphis sacchari* Zehtner (Hemiptera: Aphididae)*,* a new invasive pest threatening sorghum production in the U.S. [19, 20]*.* While the species has been present in the U.S. in sugarcane for over a century, an invasion of a new haplotype [21] absent in the country prior to the outbreaks is thought to be responsible for the eruptive population dynamics of *M. sacchari* in sorghum, which were first reported in 2013 [22, 23]. Similar to many other invasive insects [24–26], the genetic diversity of *M. sacchari* is generally low [27, 28], and sugarcane aphids colonizing sorghum in the U.S. appear to be genetically identical [29].

Current tactics used to suppress sugarcane aphids rely primarily on insecticides, but the rapid aphid population growth and high dispersion can hinder the effectiveness of the insecticides [19]. Therefore, host plant resistance has been successfully deployed to alleviate the impact of sugarcane aphids on sorghum and has proven to be an effective suppression tactic in the field [30–32]. In fact, several sorghum varieties have high levels of resistance to sugarcane aphids [22, 33]. For example, Szczepaniec [32] reported that host plant resistance in the resistant genotype was the most effective factor in population dynamics of *M. sacchari* when compared to insecticide seed treatment or planting date. Specifically, densities of aphids were 2–2.5 times greater on the susceptible sorghum compared to the resistant hybrid. Further, it has been demonstrated in the field that populations of these aphids can reach exponential rate of increase within two weeks of colonizing post-bloom sorghum [32]. While suppressive effects of host plant resistance on aphid population growth were not surprising, the differences in aphid population growth between younger sorghum and sorghum close to reproductive stage have important consequences to ecology and management of the aphid. Sugarcane aphids colonize sorghum in vegetative stages in the southern U.S., where they can overwinter on non-crop vegetation [23], while wind-aided migration is responsible for aphid colonization of sorghum in reproductive stages in the northern states (e.g., High Plains) [32]. Mechanisms underlying sorghum resistance to sugarcane aphids across sorghum phenology are not well understood, however.

The molecular mechanisms underlying plant-aphid interactions, in general, have been studied extensively using molecular mapping and QTLs screening [34–36]. Over the last few years, however, as it became apparent that transcriptional re-programming is the backbone of many plant defense responses, the research has shifted toward transcriptomic analysis of herbivore-plant interactions. Aphid-resistant cultivars respond to aphid feeding by up-regulation of diverse genes that contribute to aphid resistance [37–39]. These genes have been found to be involved in phytohormone signaling pathways, particularly salicylic acid (SA), jasmonic acid (JA), and ethylene (ET) [40–42]. Further, the production of plant secondary metabolites which are compounds that play a key role in defense and are often toxic to insects is another common induced response of plants exposed to insect herbivory [43, 44].

A comprehensive understanding of the molecular interactions between the susceptible and resistant sorghum and sugarcane aphid can provide insights into the mechanism of resistance and advance the potential to apply them to crop protection. Therefore, the goal of this research was to characterize transcriptional responses of a resistant and susceptible sorghum to herbivory by *M. sacchari.* Further, owing to the fact that these aphids can colonize sorghum at vegetative or at reproductive stages of sorghum development depending on the geographic location, we also explored if the interaction between sorghum resistance and sorghum age had an effect on gene expression and on population growth of the aphids. We hypothesized that expression of genes involved in defense pathways will be greater in the resistant and in younger sorghum than in the susceptible and in older sorghum and that the aphid population growth will reflect these differences.

## Results and discussion

### Mapping results and DEGs in resistant and susceptible sorghum exposed to aphid herbivory

**Fig. 1:**
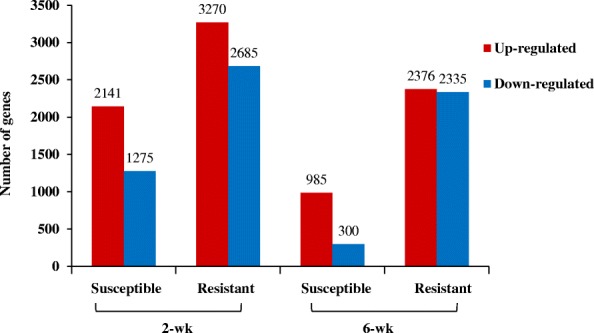
Number of DEGs in sorghum genotypes in response to sugarcane aphid herbivory at 2- and 6-wk. Columns represent number of genes up-regulated or down-regulated following exposure to aphid herbivory compared to the equivalent genotype and age treatment free of the aphids. DEGs were defined as having a fold change ≥1.9 or ≤ − 1.9 with a false discovery rate (FDR) adjusted *p*-value < 0.05

**Fig. 2:**
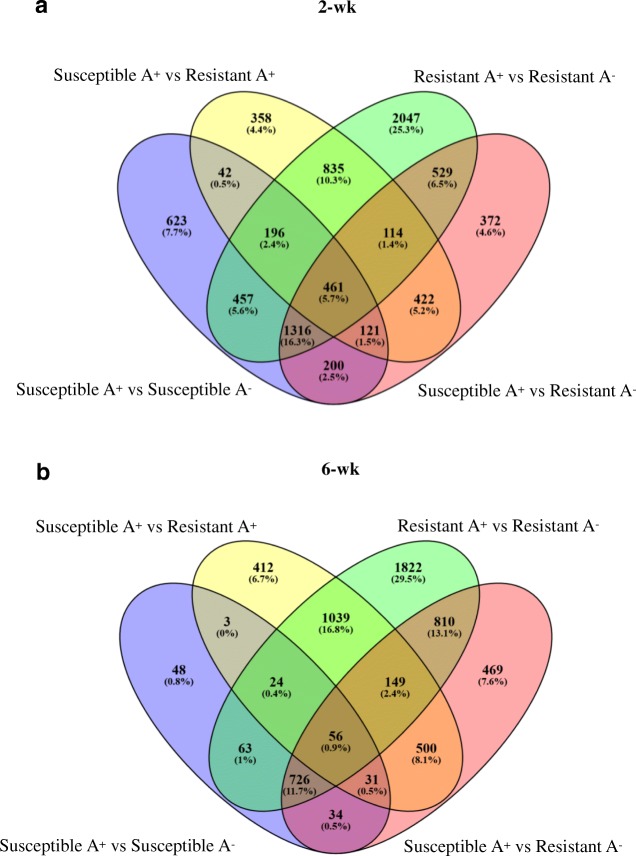
Venn diagram of DEGs in susceptible and resistant sorghum genotypes exposed to sugarcane aphid herbivory. DEGs were compared in 2-wk (**a**) and 6-wk (**b**) sorghum plants in response to aphid herbivory (A^+^) and in aphid-free (A^−^) plants. All overlapped and unique DEGs from different comparisons are shown

Sequencing of 24 libraries generated 51.6 to 82.8 million reads from individual samples, and 43.9 to 68.8 million reads were uniquely mapped to sorghum reference genome sbicolor_454 v3.1.1. On average, 91% of the reads mapped to exonic regions (Additional file 1). A higher number of DEGs was recovered from the 2-wk plants exposed to aphid herbivory compared to the 6-wk plants across genotypes. The number of DEGs was also affected by genotype such that the resistant genotype had a higher number of DEGs in response to aphid herbivory at both 2- and 6-wk post-emergence than the susceptible genotype (Fig. 1). Of the 5955 DEGs in the 2-wk resistant genotype exposed to aphid herbivory, 54.9% were up-regulated and 45% were down-regulated. On the other hand, 62.6% DEGs were up-regulated and 37.3% were down-regulated in the 2-wk susceptible sorghum in response to the aphid (Additional files 2 and 3). Fewer responses were noted in the 6-wk sorghum. Specifically, the resistant genotype up-regulated 2376 genes while the susceptible genotype up-regulated only 985 genes when aphids were feeding on the plants (Additional files 4 and 5). The overlaps between different sets of DEGs representing changes in the 2- and 6-wk susceptible and resistant genotypes in response to aphid herbivory were further illustrated in Venn diagrams (Fig. 2). In the 2-wk sorghum, 2430 DEGs were shared between both genotypes in response to aphid herbivory, while 3525 and 986 DEGs were only expressed in the resistant and susceptible genotypes, respectively. This result indicates that the resistant 2-wk old sorghum responds to the aphid feeding by eliciting a higher number of unique responses than the susceptible genotype. On the other hand, 869 DEGs were shared between the two genotypes exposed to the aphids at 6-wk post-emergence, and 3820 and 116 DEGs were uniquely expressed in resistant and susceptible genotype, respectively. The higher numbers of DEGs in the younger resistant genotype in response to *M. sacchari* indicate that these plants were undergoing significant metabolic changes. This outcome was not surprising and has been reported previously. For example, Lee et al. [37] found higher number of DEGs in resistant soybean in response to soybean aphid, *Aphis glycines* Matsumura (Hemiptera: Aphididae) in comparison to an aphid-susceptible soybean line.

### Gene ontology (GO) analysis

**Fig. 3:**
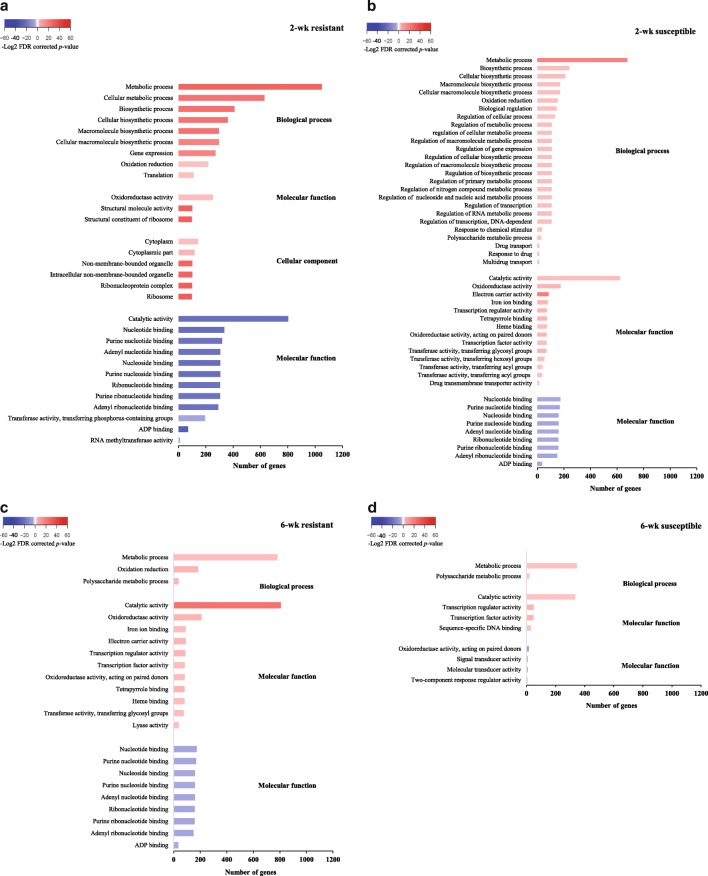
Enriched Gene Ontology (GO) terms in the two sorghum genotypes in response to aphid herbivory. GO terms were compared among the 2-wk old resistant sorghum **a**, 2-wk old susceptible sorghum **b**, 6-wk old resistant sorghum **c**, and the 6-wk old susceptible sorghum (**d**). GO terms (FDR corrected *p*-value < 0.05) with significant numbers of up-regulated or down-regulated genes were identified by contrasting gene expression in response to aphid herbivory and control (aphid-free plants). DEGs were then grouped into and biological process, molecular function, and cellular component categories. Color of the bars refers to the –log^2^ corrected *p*-value of the respective GO term. Red colored bars indicate enriched GO terms in up-regulated genes, and blue colored bars indicate enriched pathways in down-regulated genes. The darker the red color the higher statistical significance

Aphid herbivory affected a higher number of significant GO terms in the 2-wk plants than in the 6-wk plants regardless of genotype (Fig. 3). In the resistant 2-wk genotype, up-regulated genes associated with metabolic process, biosynthetic process, macromolecule process, and oxidation reduction were included in the enriched biological process GO terms. “Oxidoreductase activity” was also the prominent enriched GO term in the molecular function category in the 2-wk resistant genotype (Fig. 3a). Similarly, we noted enrichment in polysaccharide biosynthesis, carbohydrate biosynthesis, metabolic process, and oxidation reduction GO terms in up-regulated genes in the 2-wk susceptible genotype (Fig. 3b). The number of enriched GO terms in both up- and down-regulated genes was higher in the 6-wk resistant genotype (Fig. 3c) than in the 6-wk susceptible genotype (Fig. 3d). The majority of the down-regulated genes were enriched in several nucleotide binding GO terms, and it was noteworthy that “oxidoreductase activity” was the only GO term that was down-regulated in the 6-wk susceptible genotype (Fig. 3). On the other hand, genes involved in detoxification, which is one of the defenses linked to plant response to aphid herbivory [37, 45, 46] was up-regulated in both 2- and 6-wk resistant genotype and in the 2-wk susceptible genotype (Fig. 3a, b and c). The up-regulation of genes involved in oxidative-reduction process has been reported in several plants in response to herbivory, such as in barly in response to *Diuraphis noxia* Kurdjumov (Hemiptera: Aphididae) [47], in switchgrass exposed to greenbug [48], in soybean at both compatible and incompatible interactions with soybean aphid [37, 49], and in cotton in response to *Bemisia tabaci* Gennadius (Hemiptera: Aleyrodidae) [46]. Our outcomes corroborate these studies and suggest that oxidative responses that are elicited in the resistant genotype are may be the key components of sorghum resistance to sugarcane aphids.

### KEGG pathway analysis overview

**Fig. 4:**
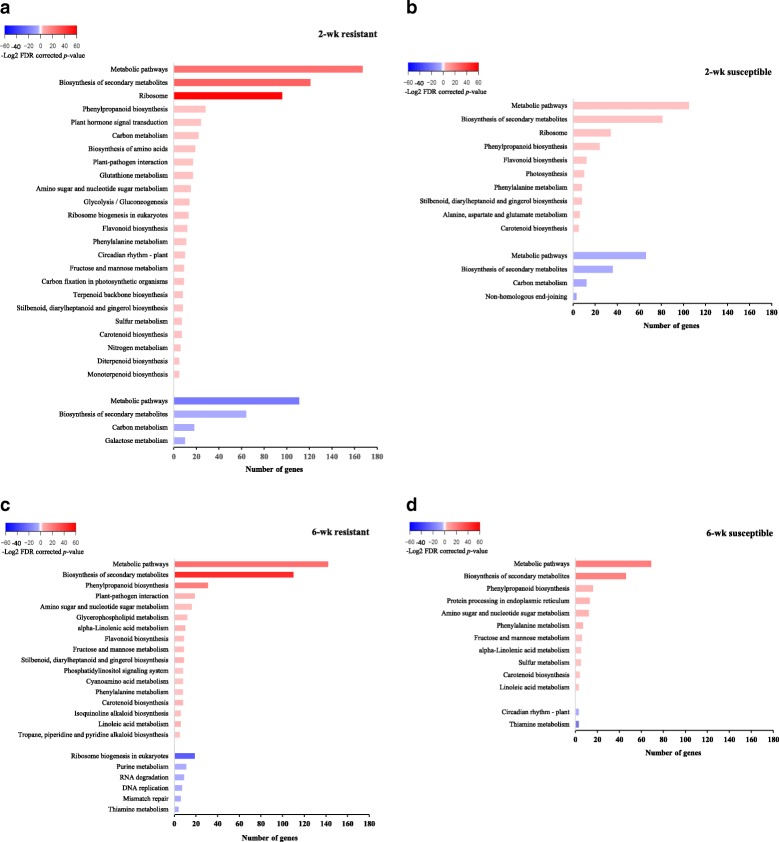
Pathways enriched in DEGs from two sorghum genotypes in response to sugarcane aphid herbivory. Pathways were compared among the 2-wk old resistant sorghum **a**, 2-wk old susceptible sorghum **b**, 6-wk old resistant sorghum **c**, and the 6-wk old susceptible sorghum (**d**) Pathways (FDR corrected *p*-value < 0.05) with significant numbers of up-regulated or down-regulated genes were identified by comparing gene expression in response to aphid herbivory and control (aphid-free plants). Color of the bars refers to the –log^2^ corrected *p*-value of the respective pathway. Red colored bars indicate enriched pathways in up-regulated genes, and blue colored bars indicate enriched pathways in down-regulated genes. The darker the red color the higher statistical significance. Length of the bar represents the number of genes within the respective GO term

Pathway analysis revealed significant effects of the genotype on a number of enriched pathways (Fig. 4). Specifically, 3270 genes that mapped to 24 pathways were up-regulated in the 2-wk resistant genotype in response to aphid herbivory, while 1275 up-regulated genes from 10 pathways were elicited in the 2-wk susceptible genotype (Fig. 4a, b). Fewer pathways were enriched in the 6-wk old sorghum.

In general, we found that a significantly higher number of pathways were up regulated rather than down-regulated in the 2-wk old plants regardless of the genotype (Fig. 4a, b). It is noteworthy that in our study plant hormone signal transduction and glutathione metabolism pathways, both shown previously to be highly relevant to plant defense [4, 50–52], were only enriched in the 2-wk resistant genotype along with a plant-pathogen interaction pathway (Fig. 4a). Induction of specific genes within these pathways suggests that their higher expression may be driving resistance to the aphids in this genotype, which appears the strongest in younger plants. In the susceptible 2-wk plants, biosynthesis of secondary metabolites, phenylpropanoid biosynthesis, and biosynthesis of flavonoids associated with plant defense and stress responses were up-regulated in response to aphid herbivory (Fig. 4b).

Aphids induced expression of genes in defense pathways in the 6-wk resistant genotype as well. These pathways included biosynthesis of secondary metabolites, phenylpropanoid biosynthesis, plant-pathogen interaction, and linoleic acid metabolism. However, the magnitude of these inductions was lower than in the younger sorghum (Fig. 4c). Biosynthesis of secondary metabolites and phenylpropanoid pathway were also enriched in the 6-wk susceptible sorghum (Fig. 4d), but the number of up-regulated genes was lower in these pathways compared with the 2-wk plants regardless of genotype. Tu et al. [53] reported that synthesis of linoleic acid in response to thrips, *Odontothrips loti* Haliday (Thysanoptera: Thripidae) increased only in the resistant alfalfa cultivar and not in the susceptible cultivar. Moreover, Ponzio et al. [54] also reported that aphid infestation in *Brassica nigra* caused the increasing of the linoleic acid. These secondary metabolites have been previously shown to be induced by herbivory in general [43, 55]. For example, Dubey et al. [56] reported the induction of transcripts related to secondary metabolite processes including phenylpropanoid and flavonoid biosynthesis in cotton exposed to *A. gossypii*. Further, Liang et al. [38] reported induction of genes involved in signal transduction, flavenoid metabolism, and plant-pathogen interactions that were likely invovled in resistance of cucumber to *A. gossypii*.

### Specific genes and pathways relevant to sorghum resistance to *M. sacchari*

Based on the GO enrichment analysis and KEGG pathway enrichment analysis, we identified multiple defense-related genes that were differentially expressed in response to aphid herbivory. These aphid-responsive genes were grouped according to their function and discussed further. Specifically, we focused on signaling related genes, which act as mediators of herbivory-induced changes that occur during the early phases of the attack [4, 46, 57, 58] and phytohormone signaling pathways. We also elaborate on pathways and genes involved in secondary metabolism and stress response, and transcription factors (TFs), which are essential for the regulation of gene expression [59].

#### Signaling-related genes: Calcium sensors, ROS, and MAPKs

A number of genes from the Ca^2+^ sensors group were up-regulated in response to aphid herbivory regardless of genotype or sorghum age. These genes encode calmodulin-like (CMLs) and calmodulin (CAM) proteins that are important in herbivore defense pathways in plants [57, 60]. We noted several genes from this family to be affected by exposure to the aphids. For example, gene *Sobic.001G393500* from the CAM gene family showed higher expression in response to aphid herbivory regardless of genotype and age, while another gene from this family, *Sobic.008G159100* was up-regulated in the 6-wk sorghum (Additional files 2, 3, 4 and 5). Further, the majority of mitogen-activated protein kinases (MAPKs) genes including LPR receptor-like, and serine/threonine-protein kinase were up-regulated in response to aphid herbivory in the resistant genotype regardless of age. The activation of MAPKs, in turn, can phosphorylate transcriptional regulators and ultimately affect gene transcription [61]. The serine/threonine-protein kinase *OXI1* gene (*Sobic.006G128200*), involved in signaling pathway by producing reactive oxygen species (ROS), was up-regulated in response to aphid across sorghum genotypes and age. The *OXI1* gene in Arabidopsis has been reported to be required for resistance to downy mildew infection [62]. Moreover, genes from the protein phosphatase 2C family (PP2C) involved in phosphorylation were also up-regulated in response to the aphid herbivory. Plant PP2Cs have been reported to be relevant to plant stress signaling [63, 64].

#### Signaling-related genes: Plant hormone signal transduction

**Fig. 5:**
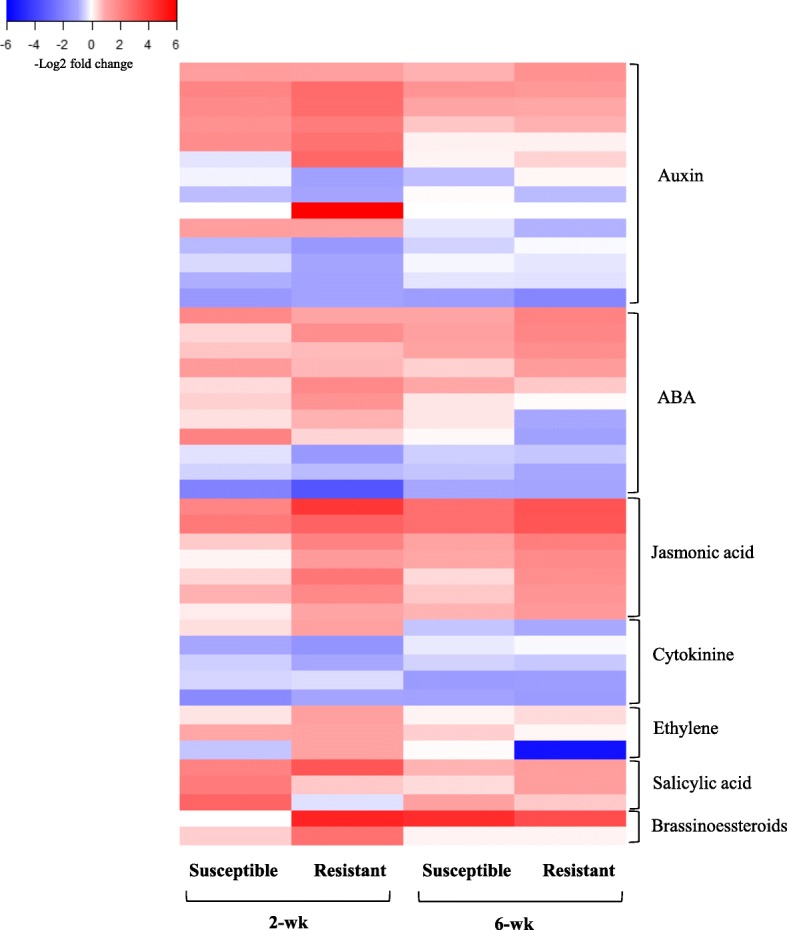
Heat map of DEGs involved in hormone transduction pathways in response to aphid herbivory. Heat map shows DEGs across two resistant and susceptible sorghum genotypes at 2- and 6-wk post-emergence. The color key represents log2-transformed fold changes and red indicate increased expression in response to aphid, while blue represents a decrease in expression

In hormone signal transduction pathways, the majority of DEGs (25 genes) were up-regulated in the 2-wk resistant genotype in response to aphid herbivory (Fig. 5). These genes belong to the SA, JA, abscisic acid (ABA), auxin, cytokinins (CKs), and ET signal transduction pathways. Among these phytohormones, salicylic acid plays an important role in mediating induced plant responses to pathogens in particular [40, 65, 66], but has been implicated in herbivore responses as well [67, 68]. In this pathway, *Sobic.001G143000* gene [*NONEXPRESSOR OF PATHOGENESIS-RELATED GENE 1* (*NPR1*)], had a 10.6- and 2.4-fold increase in expression in response to aphid herbivory in both 2- and 6-wk resistant genotype, respectively (Additional files 2 and 4). In the susceptible genotype, three *NPR1* genes were up-regulated only in the 2-wk plants exposed to the aphids (Additional file 3). The NPR1 is a SA receptor protein and the transcriptional regulation of *NPR1* is a key factor in regulation of defense signaling responses downstream of SA [69, 70].

Several DEGs in the JA signaling pathway that regulates direct and indirect plant responses against herbivores [71–73] were also induced in our experiment. These DEGs included genes from the jasmonate ZIM-domain (JAZ) family, which function as repressors of JA signal output and high levels of JAZ proteins control the JA responses by repressing their transcription factors [73, 74]. These genes had more than 2-fold change in response to aphid herbivory in the 2- and 6-wk resistant and susceptible genotype (Fig. 5). They are located up-stream of *MYC2* transcription factor and act as the negative regulator of *MYC2* function. The *MYC2* transcription factor, on the other hand, increases JA-mediated resistance to insect pests [75]. These interactions are examples of negative crosstalk between JA and SA [76, 77], and the outcomes of our experiments indicate an antagonistic interaction between these hormones in sorghum exposed to aphid herbivory. We noted the greatest expression of *NPR1* genes along with up-regulation of *JAZ* genes that act as negative regulator of the JA-signaling pathway in both 2- and 6-wk resistant and in the 2-wk susceptible genotype. On the other hand, *JAZ* genes were upregulated while *NPR1* gene expression was unaffected in the 6-wk susceptible genotype, which had the weakest overall transcriptional responses to aphid herbivory. As has been reported previously [40, 78], interactions between JA and SA are complex and can be host- [79] and herbivore-specific [80]. Moreover, it has been suggested that insects can manipulate plant defenses by modulating the JA/SA crosstalk [81–83]. Therefore, we speculate that up-regulation of *JAZ* genes may be mediated by sugarcane aphids in order to suppress host defense strategies. This was previously demonstrated by Moran, Thompson [84] who reported that green peach aphid (*Myzus persicae* Sulzer, Hemiptera: Aphididae) and greenbug aphid feeding on Arabidopsis and sorghum respectively, up-regulated SA-dependent pathway and reduced activation of JA-dependent defenses in host plants.

Abscisic acid is a plant hormone involved primarily in the perception of abiotic stresses [85–87], which can be elicited by aphid herbivory [88, 89]. Four genes in the ABA signaling cascade were up-regulated in the resistant 2-wk sorghum including *Sobic.006G279100*, *Sobic.001G350700* genes encoding SNF1-related protein kinase 2 (SnRK2, a positive regulator of ABA), and *Sobic.003G198200*, *Sobic.001G424400* genes coding for type 2C protein phosphates (PP2C, a negative regulator of ABA) (Additional file 2). Five genes in this pathway were also up-regulated in the resistant 6-wk sorhgum, including *bZIP23* transcription factor (*Sobic.004G309600*), which showed 3.2-fold induction in response to aphid herbivory (Additional file 4). Zong et al. [90] reported that *OsbZIP23* acts as a central regulator in ABA signaling, biosynthesis, and drought resistance in rice. Therefore, the effects of aphid feeding on inducing of ABA signaling are likely related to induced water stress [91, 92].

Genes involved in auxin signaling pathway were also differentially expressed in both resistant and susceptible genotypes (Fig. 5). Auxin is involved in regulation of plant growth and development, and activation of auxin signaling is usually linked to increased disease susceptibility [93, 94]. The majority of these genes were up-regulated in 2-wk sorghum regardless of genotype (Fig. 5). However, there were key differences between the resistant and susceptible 2-wk old plants. Specifically, two members of *AUX/IAA* gene family i.e. *Sobic.009G069700* and *Sobic.002G055900* that act as a transcriptional repressor were up-regulated in the 2-wk resistant genotype in response to aphid herbivory, but only *Sobic.002G055900* gene was up-regulated in the susceptible 2-wk genotype (Additional files 2 and 3). While these trends in expression are potentially important, it is not clear if these subtle transcriptional responses can explain why sugarcane aphid performance differs among genotypes.

The SA-regulated auxin homeostasis, in general, has interesting implications for the trade-off between growth and defense [95, 96] such that higher SA levels reduce the pool of active indole-3-acetic acid [97] and subsequently defense is prioritized over growth. On the other hand, in absence of pathogens, auxin-mediated suppression of SA responses can have positive consequences for plant growth [98]. In our experiment, *Sobic.002G361500* encoding *GH3–8*, an auxin-responsive gene involved in processes that halt accumulation of free IAA, showed a 6.6- and 3.9-fold increases in expression in response to aphid herbivory in 2-wk resistant and susceptible genotype, respectively (Additional files 2 and 3). This suggests that up-regulation of these genes may have triggered a change in allocation of resources from growth to defense and conferred greater resistance to the aphids in the younger plants. It has been also reported that auxin-regulated genes such as *AUX/IAA* and *GH,* are involved in an inhibitory feedback loop that maintains cellular auxin homeostasis that is required for adjusting auxin concentration for the optimal plant growth and development [99–101]. Induction of these genes in the 2-wk sorghum regardless of genotype but not in the 6-wk old sorghum suggests that the younger plants may be diverting their resources from primary to secondary metabolism thus increasing their resistance to the herbivore. However, more research is needed to unequivocally link these transcriptional responses to decreased aphid performance in the younger and resistant sorghum.

Cytokinins are plant hormones that act as key regulators of plant growth-defense trade-off and are also involved in plant-biotic interactions [98, 102]. We found a number of significantly induced genes in cytokinine signaling pathway, several of which were induced in the absence of aphids and suppressed when aphids were feeding on the plants. Specifically, in the 2-wk aphid-free resistant sorghum, three positive cytokinine regulator genes (*Sobic.009G202900*, *Sobic.003G292600*, and *Sobic.004G330900*) were up-regulated, indicating that plants were regulating their growth processes actively (Additional file 2). On the other hand, *Sobic.006G090300* gene, a two-component response regulator *ORR1* that acts as a negative regulator of cytokinine [98] was up-regulated in 2-wk resistant genotype exposed to aphid herbivory, suggesting that the younger resistant plants were suppressing their growth-related processes (Additional file 2). This outcome provides another example of the younger resistant sorghum switching from growth to defense in response to the aphids and highlights the lack of parallel responses in the susceptible sorghum. These results indicate a potential trade-off between growth and defense, which are both metabolically costly processes that usually cannot be carried out concurrently at high levels [96].

Ethylene is one of induced defense related phytohormones that is produced in response to multiple stresses [103, 104]. Ethylene expression decreases with plant age, with a marked decline at the onset of reproduction associated with a significant reduction in the ability of plants to induce defenses [105]. This was consistent with our results – we found ethylene gene expression only in the younger plants. In ethylene signaling pathway, two genes i.e. *Sobic.007G210700* and *Sobic.009G050400*, involved in the ethylene pathway were both up-regulated in the resistant 2-wk genotype in response to aphid and only *Sobic.007G210700* was up-regulated in the susceptible 2-wk genotype (Additional files 2 and 3). The release of ethylene in response to aphid herbivory has been reported in other host plant species [2, 106, 107].

#### Secondary metabolites related to oxidative stress response genes

**Fig. 6:**
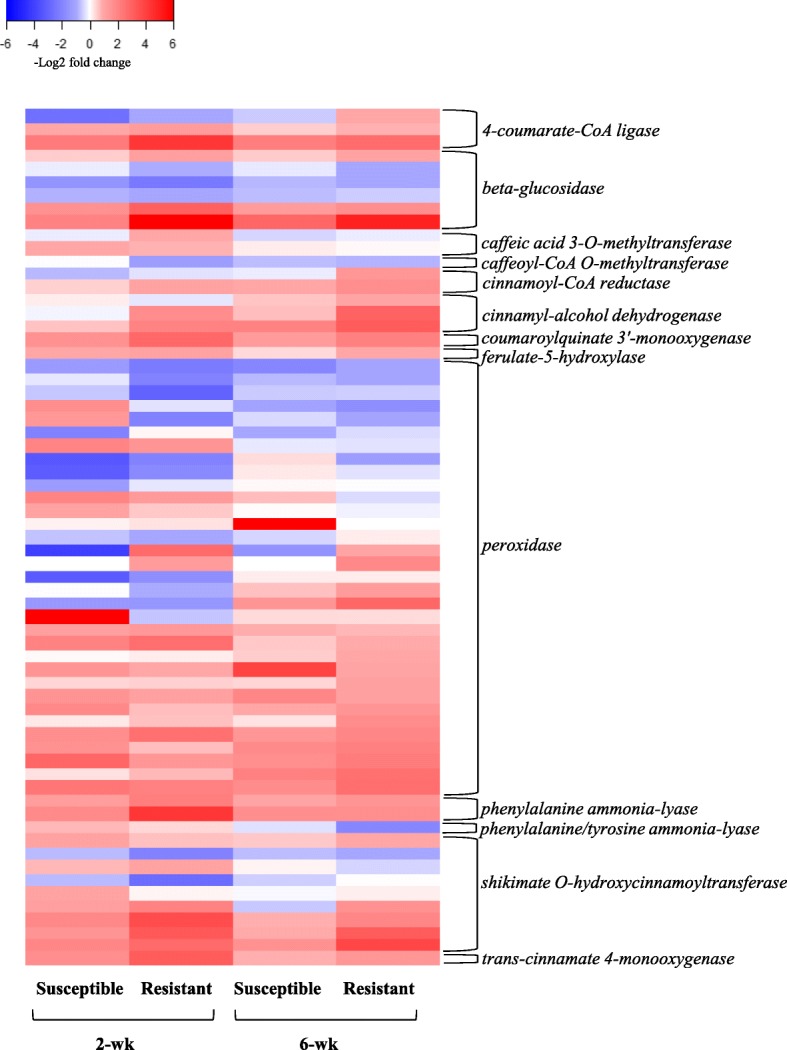
Heat map of DEGs involved in phenylpropanoid biosynthesis pathway. Heat map presents DEGs across two resistant and susceptible sorghum genotypes at 2- and 6-wk post emergence. The color key represents log2-transformed fold changes and red indicate increased expression in response to aphid, while blue represents a decrease in expression

A number of DEGs affected by aphid herbivory in our study are involved in metabolite synthesis through phenylpropanoid and flavonoid biosynthesis pathways. In addition, several other genes that were induced regulate plant responses to oxidative stress caused by herbivory. A number of genes in phenylpropanoid biosynthesis pathway showed differential expression in both susceptible and resistant genotypes exposed to the aphids (Fig. 6). Specifically, two genes *Sobic.004G220400* and *Sobic.004220700*, both encoding phenylalanine ammonia-lyase (PAL) showed more than 2-fold increase in response to aphid herbivory in both the 2- and 6-wk resistant and susceptible genotypes (Additional files 2, 3, 4, 5). Phenylalanine ammonia-lyase catalyzes the first step of the phenylpropanoid pathway, which is a key reaction in the control of lignin, flavonoid and salicylic acid biosynthesis [108]. Lv et al. [108] reported that expression of *PAL* was significantly increased in cotton and corn seedlings damaged by mechanical wounding or by cotton aphid and corn borer, *Ostrinia furnacalis* Guenée (Lepidoptera: Crambidae) herbivory. Further, Chaman et al. [109] reported a positive relationship between SA concentration, *PAL* activity, and resistance of barley to aphid infestations. Therefore, induction of these genes across sorghum genotypes and age in our experiment indicates that they play a key role in sorghum response to aphid herbivory regardless of sorghum age or genotype.

Genes that were involved in response to oxidative stress belong to *glutathione S-transferase*, *L-ascorbate peroxidase*, *peroxidase*, and *cytochrome P450* gene family. The up-regulation of a number of genes involved in glutathione metabolism pathway was mostly found in the resistant 2-wk sorghum in response to aphid herbivory (Additional file 2). It has been proposed that the activation of glutathione metabolism is a natural cell response to stress that can be induced by aphids in general [56, 110], and the increase in expression of enzymes involved in glutathione synthesis is correlated with resistance to various biotic challenges [111] and in the detoxification of ROS [112]. Further, among the DEGs involved in phenylpropanoid pathway in our experiment, a higher number of genes encoding peroxidase showed differential expression in response to aphid herbivory in younger plants in comparison with older ones regardless of genotype. Peroxidases not only code for ROS-detoxifying enzymes but are also involved in oxidative signal transduction, regulating the redox and Ca^2+^ homeostasis as well as the expression of defense genes [113–115]. Higher expression of peroxidases has been reported previously in barley exposed to *Diuraphis noxia* (Hemiptera: Aphididae) [47], and in tomato fed upon by *Macrosyphum euphorbiae* (Hemiptera: Aphididae) [58]. Likewise, the *cytochrome P450* superfamily genes were another abundant gene family that was induced in response to oxidative stress imposed by the aphids in our study. This gene family plays a crucial role in plant defense via multiple biosynthetic and detoxification pathways [116]. Prochaska et al. [39], for example, reported that genes from *cytochrome P450* family were up-regulated in the resistant genotype of soybean in response to aphid herbivory. Notably, we found these genes to be induced the strongest in the 2-wk resistant sorghum exposed to sugarcane aphids (Additional file 2), which may explain the superior host plant resistance observed in this genotype shortly after emergence.

### DEGs encoding transcription factors

**Fig. 7:**
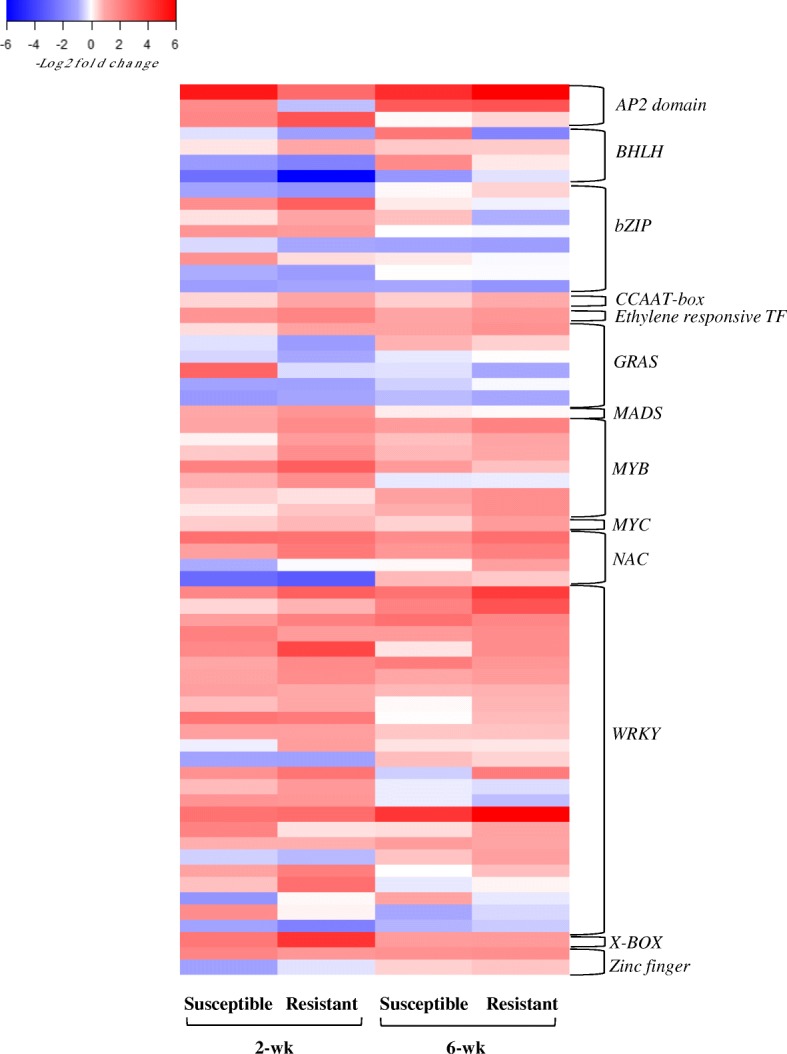
Heat map of differentially expressed transcription factors. Heat map shows DEGs across two resistant and susceptible sorghum genotypes at 2- and 6-wk post-emergence. The color key represents log2-transformed fold changes and red indicate increased expression in response to aphid, while blue represents a decrease in expression

Our results indicated differential expression of 71 genes encoding transcription factors (TFs) including *WRKY*, *MYB*, *bHLH*, *NAC*, and *AP2/ERF*. The *WRKY* family of TFs is unique to plants and is involved in regulation of defense responses to pathogens and insects [117, 118]. The involvement of selected *WRKY* family in response to biotic stresses has been reported in Arabidopsis [119, 120], and we also tracked the expression of the orthologs of these genes in sorghum. Specifically, we found the highest number of differentially expressed TFs (52) in the resistant genotype and in the younger plants, and 18 of these TFs belonged to the *WRKY* family. In the 6-wk resistant genotype, a total of 42 TFs were up regulated in response to aphid herbivory, and 13 of these were *WRKY* TFs. A comparable number of *WRKY* genes were up-regulated in response to aphid herbivory in the 2- and 6-wk susceptible plants (Fig. 7). It is noteworthy that four *WRKY* transcription factors were found to be expressed exclusively in the 2-wk resistant genotype exposed to the aphids, including *WRKY1* (*Sobic.001G095500*), *WRKY19* (*Sobic.009G238200*), *WRKY28 (Sobic.003G199400*) and *WRKY72* (*Sobic.005G117400*) (Additional file 2). The involvement of the homologs of these genes in hormone signaling pathways and response to abiotic stresses has been reported in Arabidopsis [42, 78, 119, 121]. For example, the homolog of *WRKY19* in Arabidopsis, i.e., *AT4G11070* has been shown to be a key regulator of cross-talk between SA and JA signaling pathways [122]. Our outcomes corroborate these reports – the sorghum orthologs of *AtWRKY70* i.e. *Sobic.003G337900*, and *Sobic.008G060300* were only expressed in response to aphid herbivory in 2-wk plants regardless of genotype, with higher expression in the resistant genotype. Similarly, the *AtWRKY40* ortholog in sorghum, i.e. *Sobic.004G065900*, was up-regulated in response to aphid herbivory regardless of genotype and age. The up-regulation of *AtWRKY40* has been reported in Arabidopsis in response to cabbage aphid, *Brevicoryne brassica* (Hemiptera: Aphididae) [120]. Three TFs with AP2 domain i.e., *Sobic.002G415600*, *Sobic.003G380900*, and *Sobic.004G310600*, prominent TF regulators of host defense [123, 124], were up-regulated in response to aphid herbivory across genotypes and sorghum age. Other types of TFs identified among the DEGs were *bZIP*, *bHLH*, *MYB, NAC*, *zinc finger,* several of which have been reported to be important for plant adaptive response to biotic stress [125, 126].

### Real-time quantitative RT-PCR (RT-qPCR) of selected DEGs

All genes selected for validation of RNA-seq data showed a similar expression trend (up-regulation or down-regulation) in the RT-qPCR analysis. High correlations (all above R^2^ > 0.70) were found between RNA-seq and RT-qPCR results (Additional file 6), indicating that the measured changes in gene expression detected by RNA-seq reflected the actual transcriptome differences between the samples.

### Aphid population growth on resistant and susceptible sorghum 2- and 6-wk post-emergence

**Fig. 8:**
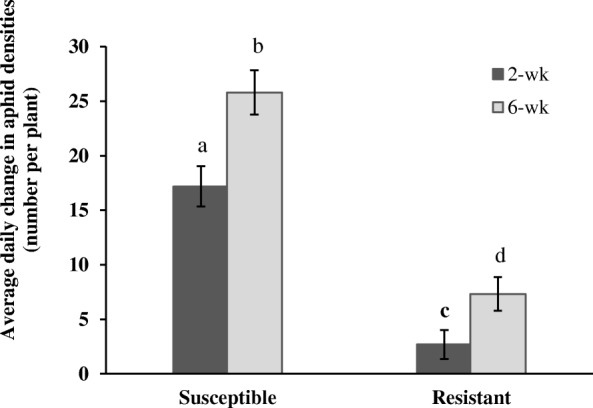
Impact of sorghum genotype and age on average daily change of aphid density. Daily change in aphid density was measured on susceptible and resistant sorghum 2- and 6-wk post emergence. Bars mark means ±1 SEM and means with different letters differed significantly (*P* ≤ 0.05)

Sorghum variety and growth stage had a significant effect on the mean daily change in *M. sacchari* densities (Fig. 8). The average change in aphid densities in susceptible sorghum was significantly greater than in resistant sorghum (*F*_1,32_ = 92.87; *p-*value < 0.001), and aphid populations increased faster in the 6-wk old sorghum than in the 2-wk old sorghum (*F*_1,32_ = 15.01; *p-*value < 0.001). However, there was no interactive effect of these factors on the average change in aphid densities (*F*_1,32_ = 1.36; *p-*value = 0.253). Aphid populations grew the slowest in the young resistant sorghum, and their numbers increased the most rapidly in the older susceptible sorghum. Rapid population growth of *M. sacchari* in susceptible sorghum in reproductive stages has been reported previously [32], and our outcomes corroborate those findings. Further, these results indicate that the robust transcriptional response to aphid herbivory in the younger resistant genotype is phenotypically reflected in the significantly diminished aphid performance on these plants.

## Conclusions

The host plant resistance reported previously for the resistant genotype and manifested in significantly lower aphid densities in the greenhouse and field experiments [32] was reflected at the transcriptome level in our experiment. Specifically, transcriptome analysis of the susceptible and resistant sorghum genotypes revealed a suite of genes and pathways elicited in response to *M. sacchari* herbivory, particularly in the resistant and younger sorghum. These differentially expressed genes were involved in diverse processes including signaling, metabolism, and oxidative stress. We detected a higher number of DEGs and expression of a higher number of defense related genes in the resistant genotype in response to aphid herbivory than in the susceptible genotype. Further, the 2-wk old resistant genotype, in particular, mounted the most robust response to *M. sachari* feeding, and several genes and pathways provided insight into the mechanisms of aphid resistance.

The enriched GO terms and pathways highlighted the general differences between the two genotypes in response to aphid herbivory. The oxidation reduction GO term was highly enriched in the resistant genotype compared to the susceptible genotype. We also found that pathways coding for secondary metabolites, plant hormone signal transduction, plant-pathogen interaction, and glutathione metabolism were over-expressed in the resistant genotype. The resistant genotype also activated more genes involved in SA, JA, ET, and ABA signaling pathways. Our results suggested a potential cross-talk between SA and JA in the resistant genotype such that up-regulation of SA pathway genes negatively affected JA pathways genes through higher expression of JAZ genes that act as a negative regulator of JA biosynthesis pathway genes. Aphid herbivory also affected the balance between growth and defense related genes to the benefit of defense. Similar differences were noted when gene expression was compared between the 2-wk and 6-wk old sorghum – the number of DEGs, enriched GO terms, and significantly affected pathways were higher in the 2-wk old plants than in the 6-wk old sorghum. On the other hand, the lower resistance of older plants may be related to the developmental stage of these plants. Sorghum at 6-wk after planting are usually at panicle initiation stage, and are especially sensitive to biotic and abiotic stress during this phase [127].

In conclusion, sugarcane aphid herbivory elicited the most robust transcriptional changes in the younger resistant genotype, and several pathways and specific genes provide insights into the mechanisms underlying host plant resistance to this invasive insect. Despite the apparent cross talk between SA and JA elicited by aphid herbivory, the younger resistant plants regulated their responses to abiotic stress, diverted resources to defense over growth, and induced strong responses to oxidative stress at the highest level compared to all other treatments. Lack of parallel responses in the older sorghum plants has relevant implications for aphid-sorghum interactions, and suggests lower host plant resistance in sorghum colonized at the onset of the reproductive stage. Further, unique induction of four *WRKY* genes only in the 2-wk resistant genotype highlights the likely importance of these TFs in the superior resistance of these plants to aphids, and should be considered in future studies. Further research should explore functional role of genes we found to be induced by *M. sacchari* to further clarify their impact on aphid-plant interactions.

## Methods

### Sorghum growth and *M. sacchari* colony

All experiments were conducted at the Texas A&M AgriLife Research greenhouse complex located at the Plant Stress Laboratory in Bushland, TX. Two sorghum genotypes, aphid-susceptible (DKS 44–20, Dekalb ®, Dekalb, IL), and aphid-resistant (DKS 37–07, Dekalb ®, Dekalb, IL) at 2- and 6-wk post-emergence grown under a long-day photoperiod regime were used in this study. The two genotypes were grown under controlled greenhouse conditions with a temperature range of 21 ± 3 °C [62] to 30 ± 3 °C [128]. Plants were grown in pots containing Redi-earth plug and seedling soil mix (Sun Grow Horticulture Inc., Agawam, MA) in 60 × 60 × 60 cm insect-proof rearing tents (MegaView Science Education Services Co., Taipei, Taiwan). Plants assigned to the 6-wk treatment were grown in 1 × 1 × 1 m cages constructed from PVC pipes and insect-proof mesh.

Sugarcane aphid colony originated from aphids collected from sorghum growing on a research farm affiliated with Texas A&M AgriLife Research in Bushland, TX, and no permits were required to enter the land or collect the aphids. The colony was maintained in the greenhouse for one year prior to the experiment. In order to confirm that the colony was genetically homogeneous, universal DNA primers were used to amplify a 710 bp fragment of the mitochondrial cytochrome c oxidase subunit I gene (*COI*) following Vrijenhoek [129] methods. Two samples from two cages harboring the colony were collected for this analysis. The PCR products were purified using QIAGEN MinElute PCR purification kit (QIAGEN, Valencia, CA, USA) and sequenced on ABI 3130xl genetic analyzer by the Institute for Plant Genomics and Biotechnology at Texas A&M University. The sequencing data from four samples were compared with each other using EMBOSS Needle [pairwise sequence alignment (Nucleotide)] (http://www.ebi.ac.uk/Tools/psa/emboss_needle/nucleotide.html). There was no variation within and between a population that confirmed the integrity of the aphid colony. Further, the *COI* sequencing confirmed that the aphids were of different haplotype than previously described in sugarcane in the U.S. [27].

### Sample collection, RNA isolation, and sequencing

The experiment was a 2 × 2 × 2 factorial design with two levels of the sorghum genotype factor (susceptible, resistant), two levels of the sorghum age (2-wk, 6-wk), and two levels of aphid herbivory (present, absent). An individual pot was considered a biological replicate and three biological replications and three technical replications (i.e. individual plant in each pot) were included in this experiment. Half of the susceptible and half of the resistant plants were randomly assigned to one of the two sorghum age treatments and the herbivory treatment, which consisted of exposing two leaves in each plant to 15 aphids for 24 h. The entire above-ground tissue of each 2-wk plant was collected for RNA-seq. Six-wk old plants were exposed to aphids in the same manner, with the exception of tissue collection for RNA extraction – only leaves exposed to the aphids and not entire plants were collected. Owing to the extensive size of the 6-wk old sorghum that surpassed 50 cm in height and had more than 10 fully unfurled leaves only the tissue that was directly exposed to aphid herbivory was collected and used in RNA extractions. Aphids on the 2-wk old plants, on the other hand, were not confined to the single leaf to which they were introduced, and hence the entire plant (10–15 cm in height with three leaves) was collected. Samples were immediately frozen in liquid nitrogen (LN_2_) and stored at − 80 °C until further sample processing.

Frozen leaf samples were ground under LN_2_ into a fine powder using a mortar and pestle. RNA was extracted from 100 mg of frozen ground tissue using the miRNeasy Mini Kit (Qiagen, Valencia, CA, USA) and subsequently treated with DNase Max Kit (Qiagen, Valencia, CA, USA). RNA quality and quantity were assessed using the NanoVue Plus spectrophotometer (GE, Healthcare, Piscataway, NJ, USA). Three equimolar RNA samples extracted from three technical replicates for each biological replicate were pooled after extraction for RNA-seq template preparation. The quality of each pooled RNA sample was assessed with an Agilent 2100 Bioanalyzer (Agilent Technologies, Santa Clara, CA, USA) prior to RNA-seq template preparation by Texas AgriLife Research Genomic and Bioinformatics Services. Paired-end (PE) sequencing was performed on an Illumina HiSeq4000 using a 75 bp paired-end strategy on three biological replicates. RNA-seq template preparation and sequencing was completed at Texas AgriLife Research Genomics and Bioinformatics Center. The quality of RNA-seq reads was assessed using FastQC (http://www.bioinformatics.babraham.ac.uk/projects/fastqc/).

### Gene expression analysis

Sequence reads were imported into the CLC Genomics Workbench version 11 (Qiagen, Valencia, CA, USA) and mapped to the *S. bicolor* reference genome [Sbicolor_454 v3.1.1, www.phytozome.jgi.doe.gov, [130]. Based on the total read counts for each annotated gene, differential gene expression analyses were conducted using the Empirical Analysis of DGE tool, which implements the ‘Exact Test’ for two-group comparisons [131]. The transcriptional response of sorghum plants at 2- and 6-wk post-emergence and exposed to aphid herbivory was compared to aphid-free plants (the control) to identify DEGs. Differentially expressed genes were defined as having a fold change ≥1.9 or ≤ − 1.9 with a false discovery rate (FDR) corrected *p*-value < 0.05. For functional annotation, GO analysis was performed using AgriGO gene ontology analysis tools [132] to determine overrepresented GO categories in the up- and down-regulated DEGs, and significantly enriched GO terms were identified. Pathway analysis was performed using the KEGG database [133] to underline the pathways to which the up- and down-regulated DEGs contribute, and pathway enrichment analysis completed using the KOBAS server (version v.3) [134]. The overlaps between different sets of DEGs were generated with VENNY.2.1 (http://bioinfogp.cnb.csic.es/tools/venny/).

### RT-qPCR analysis of selected DEGs

Five DEGs involved in plant-hormone signal transduction pathway were selected for validation of RNA-seq data using RT-qPCR. The same RNA samples from three biological replicates that were used for the sequencing were used for the validation of RNA-seq data. The total RNA was reverse-transcribed using Omniscript RT Kit (QIAGEN, Valencia, CA, USA). Primer pairs were designed using NCBI Primer-BLAST (http://www.ncbi.nlm. nih.gov/tools/primer-blast/), which are listed in Additional file 7. The RT-qPCR reaction was performed in a total volume of 20 μl, containing 1 μl of diluted cDNA, 0.5 μl of reverse and forward primers, 8 μl of ddH2O, 10 μl of SYBR Green Master Mix (Applied Biosystems, Foster City, CA, USA). Samples were run on a Applied Biosystems ViiA 7 Real-Time PCR system (Applied Biosystems, Foster City, CA, USA) according to the standard protocol as follows: 2 min at 50 °C, 10 min at 95 °C, followed by 40 cycles of 15 s at 95 °C for and 1 min at 60 °C. A melt curve was generated by the end of each PCR reaction to verify the formation of single peak and to exclude the possibility of primer dimer and non-specific product formation. All reactions were performed in duplicate, including the non-template control reactions. The relative quantitation of gene expression of RT-qPCR was measured via the 2^−ΔΔCt^ method [135], with *CYP* (*Cyclophilin/Peptidylprolyl Isomerase*) gene as the endogenous reference gene that showed the highest stability among three tested reference genes. The correlation between the RNA-seq data and the RT-qPCR results was determined by Pearson’s correlation coefficient.

### Aphid population growth evaluation

In another set of experiments, differences in aphid densities on 2- and 6-wk susceptible and resistant sorghum genotypes was evaluated in early spring 2017. Experiments were conducted in the greenhouse following the protocol described above (Sorghum growth and *M. sacchari* colony). The experimental design was a randomized complete design with two levels of sorghum growth stage (2- and 6-wk old sorghum), and two levels of the sorghum variety factor (Susceptible – DKS 44–20, and Resistant – DKS 37–07). The entire experiment was conducted twice (time block) and each of the treatment combinations was replicated a total of nine times across the two time blocks (*n* = 36). When sorghum reached the assigned growth stage, 15 sugarcane aphids were moved using a paintbrush and clippings of infested sorghum onto two leaves on each of the plants. The aphids were counted and replenished as needed 1 h following the introductions, and aphid densities following a 24 h acclimation period were used as the initial densities. Aphids were counted on entire plants every three days thereafter for 14 d. The mean daily change in aphid densities per plant was the response variable recorded and analyzed.

### Statistical analyses for greenhouse evaluation

The effects of sorghum age and variety on the average daily change in aphid densities was analyzed using two-way ANOVA with sorghum age and variety combination as a fixed factor and time block as a random factor. Owing to lack of a significant interaction between treatments and time block data from the two greenhouse experiments were combined. Effects of sorghum variety, age, and the interaction between the two factors were included in the analyses, and means separation tests were performed were appropriate with a Bonferroni adjustment. All analyses were performed in R [136].

## Additional files

Additional file 1: Summary of RNA-seq reads from susceptible and resistant sorghum genotypes mapped to the sorghum genome (Sbicolor_454 version v.3.1.1). Unique RNA-seq reads mapping to exons, introns, and intergenic regions are shown as the percentage of total reads distributed to these annotated regions of the sorghum genome. (DOCX 23 kb)

Additional file 2: Differentially expressed genes (DEGs) in 2-wk resistant sorghum genotype in response to aphid herbivory. DEGs were defined as having a fold change ≥1.9 or ≤ − 1.9 with a false discovery rate (FDR) adjusted *p*-value < 0.05. (XLSX 431 kb)

Additional file 3: Differentially expressed genes (DEGs) in 2-wk susceptible sorghum genotype in response to aphid herbivory. DEGs were defined as having a fold change ≥1.9 or ≤ − 1.9 with a false discovery rate (FDR) adjusted *p*-value < 0.05. (XLSX 258 kb)

Additional file 4: Differentially expressed genes (DEGs) in 6-wk resistant sorghum genotype in response to aphid herbivory. DEGs were defined as having a fold change ≥1.9 or ≤ − 1.9 with a false discovery rate (FDR) adjusted *p*-value < 0.05. (XLSX 339 kb)

Additional file 5: Differentially expressed genes (DEGs) in 6-wk susceptible sorghum genotype in response to aphid herbivory. DEGs were defined as having a fold change ≥1.9 or ≤ − 1.9 with a false discovery rate (FDR) adjusted *p*-value < 0.05. (XLSX 106 kb)

Additional file 6: Real-time quantitative RT-PCR (RT-qPCR) validation of gene expression changes detected by RNA-seq. Data are shown as a fold change of the mean expression levels from three biological replicates. The correlation between the RNA-seq data and the RT-qPCR results is shown by Pearson’s correlation coefficient (R^2^). (PDF 140 kb)

Additional file 7: Primers sequences used for RT-qPCR amplification of the five differentially expressed genes selected for validation. (DOCX 16 kb)
