# Supplementary material for: Effects of sugarcane aphid herbivory on transcriptional responses of resistant and susceptible sorghum
Source: BMC Genomics. 2018 Oct 26;19:774. doi: 10.1186/s12864-018-5095-x (PMC6204049; doi:10.1186/s12864-018-5095-x)
Supplement: Supplementary file 1 — Summary of RNA-seq reads from susceptible and resistant sorghum genotypes mapped to the sorghum genome (Sbicolor_454 version v.3.1.1). Unique RNA-seq reads mapping to exons, introns, and intergenic regions are shown as the percentage of total reads distributed to these annotated regions of the sorghum genome. (DOCX 23 kb) [file 12864_2018_5095_MOESM1_ESM.docx]

## **Additional file 1: Summary of RNA-seq reads from susceptible and resistant sorghum genotypes mapped to the sorghum genome (Sbicolor_454 version v.3.1.1).**

| **Genotype** | **Treatment** | **Age**  **(week)** | **Rep number** | **Total reads (M)** | **Mapped reads (M)** | **Exonic reads (%)** | **Intronic reads (%)** | **Intergenic reads (%)** |
| --- | --- | --- | --- | --- | --- | --- | --- | --- |
| **Susceptible** | Control | 2 | 1 | 64.69 | 54.24 | 88.64 | 5.17 | 6.19 |
| **Susceptible** | Control | 2 | 2 | 73.62 | 62.97 | 91.3 | 4.54 | 4.16 |
| **Susceptible** | Control | 2 | 3 | 55.66 | 47.40 | 91.42 | 4.47 | 4.11 |
| **Mean ± sd** |  |  |  | 64.65 ± 8.98 | 54.87 ± 7.80 | 90.45 ± 1.57 | 4.72 ± 0.38 | 4.11 ± 4.82 |
| **Susceptible** | +Aphid | 2 | 1 | 58.4 | 48.27 | 93.18 | 3.85 | 2.97 |
| **Susceptible** | +Aphid | 2 | 2 | 51.06 | 43.94 | 92.59 | 4.28 | 3.13 |
| **Susceptible** | +Aphid | 2 | 3 | 61.65 | 51.56 | 92.08 | 4.47 | 3.45 |
| **Mean ± sd** |  |  |  | 57.03 ± 5.42 | 47.92 ± 3.82 | 92.61 ± 0.55 | 4.2 ± 0.31 | 3.18 ± 0.24 |
| **Resistant** | Control | 2 | 1 | 60.47 | 50.58 | 92.00 | 4.58 | 3.42 |
| **Resistant** | Control | 2 | 2 | 58.02 | 49.24 | 92.26 | 4.54 | 3.21 |
| **Resistant** | Control | 2 | 3 | 63.86 | 54.23 | 92.5 | 4.11 | 3.39 |
| **Mean ± sd** |  |  |  | 60.78 ± 2.93 | 51.35 ± 2.58 | 92.25 ± 0.25 | 4.41 ± 0.26 | 3.34 ± 0.11 |
| **Resistant** | +Aphid | 2 | 1 | 67.93 | 57.37 | 92.23 | 4.42 | 3.35 |
| **Resistant** | +Aphid | 2 | 2 | 62.36 | 54.80 | 93.12 | 4.15 | 2.73 |
| **Resistant** | +Aphid | 2 | 3 | 63.02 | 53.26 | 92.79 | 4.05 | 3.16 |
| **Mean ± sd** |  |  |  | 64.43 ± 3.04 | 55.14 ± 2.07 | 92.71 ± 0.44 | 4.20 ± 0.19 | 3.08 ± 0.31 |
| **Susceptible** | Control | 6 | 1 | 52.17 | 45.25 | 90.53 | 5.39 | 4.08 |
| **Susceptible** | Control | 6 | 2 | 64.13 | 54.46 | 89.80 | 5.31 | 4.89 |
| **Susceptible** | Control | 6 | 3 | 53.23 | 46.80 | 91.57 | 4.99 | 3.43 |
| **Mean ± sd** |  |  |  | 56.51 ± 6.62 | 48.83 ± 4.93 | 90.63 ± 0.88 | 5.23 ± 0.21 | 4.13 ± 0.73 |
| **Susceptible** | +Aphid | 6 | 1 | 67.17 | 56.41 | 90.02 | 5.67 | 4.32 |
| **Susceptible** | +Aphid | 6 | 2 | 57.03 | 48.58 | 90.93 | 5.31 | 3.76 |
| **Susceptible** | +Aphid | 6 | 3 | 60.48 | 51.73 | 90.34 | 5.41 | 4.25 |
| **Mean ± sd** |  |  |  | 61.56 ± 5.15 | 52.24 ± 3.93 | 90.43 ± 0.46 | 5.46 ± 0.18 | 4.11 ± 0.30 |

| **Genotype** | **Treatment** | **Age**  **(week)** | **Rep number** | **Total reads (M)** | **Mapped reads (M)** | **Exonic reads (%)** | **Intronic reads (%)** | **Intergenic reads (%)** |
| --- | --- | --- | --- | --- | --- | --- | --- | --- |
| **Resistant** | Control | 6 | 1 | 59.25 | 50.14 | 91.00 | 5.15 | 3.85 |
| **Resistant** | Control | 6 | 2 | 82.81 | 68.86 | 90.32 | 5.56 | 4.12 |
| **Resistant** | Control | 6 | 3 | 59.41 | 49.71 | 89.87 | 5.45 | 4.67 |
| **Mean ± sd** |  |  |  | 67.15 ± 13.55 | 56.23 ± 10.93 | 90.39 ± 0.56 | 5.38 ± 0.21 | 4.21 ± 0.41 |
| **Resistant** | +Aphid | 6 | 1 | 66.00 | 56.39 | 92.17 | 4.76 | 3.08 |
| **Resistant** | +Aphid | 6 | 2 | 67.98 | 54.83 | 90.77 | 4.96 | 4.27 |
| **Resistant** | +Aphid | 6 | 3 | 61.49 | 52.7 | 91.29 | 4.99 | 3.72 |
| **Mean ± sd** |  |  |  | 65.15 ± 3.32 | 54.64 ± 1.85 | 91.41 ± 0.70 | 4.90 ± 0.12 | 3.67 ± 2.20 |

## Unique RNA-seq reads mapping to exons, introns, and intergenic regions are shown as the percentage of total reads distributed to these annotated regions of the sorghum genome.
