# Supplementary figures and images for: Effects of sugarcane aphid herbivory on transcriptional responses of resistant and susceptible sorghum
Source: BMC Genomics. 2018 Oct 26;19:774. doi: 10.1186/s12864-018-5095-x (PMC6204049; doi:10.1186/s12864-018-5095-x)

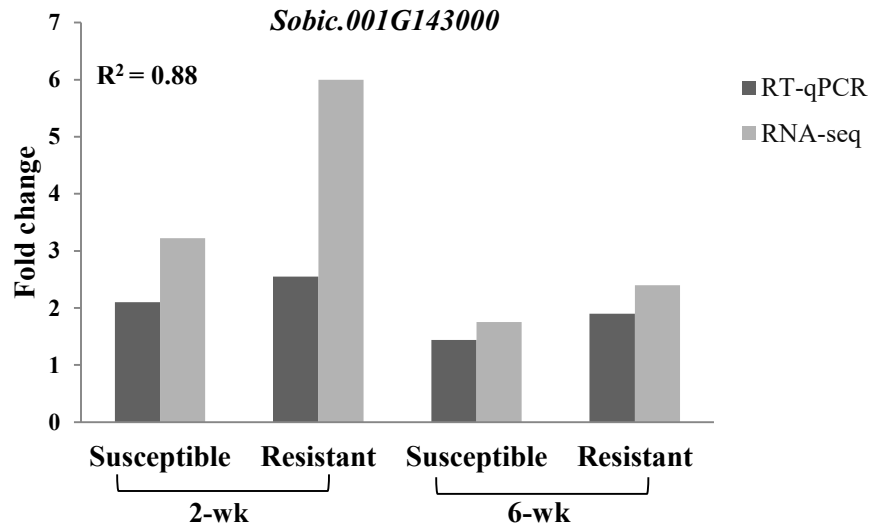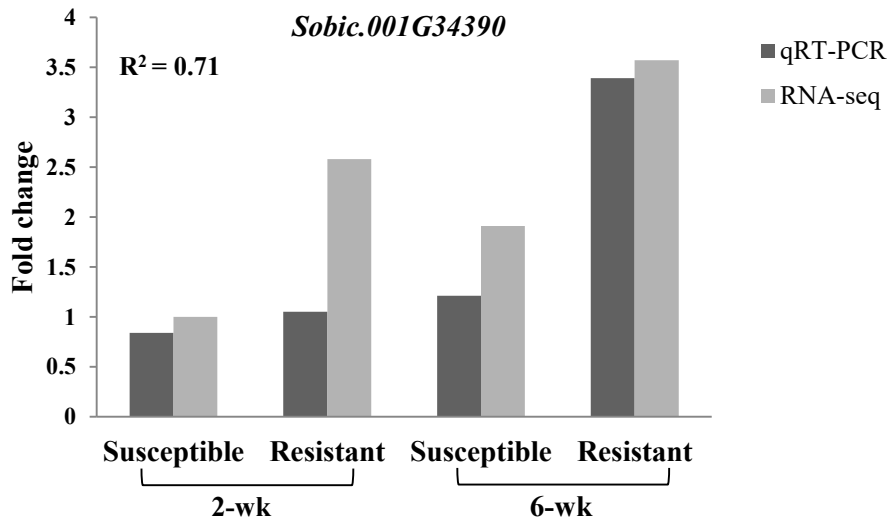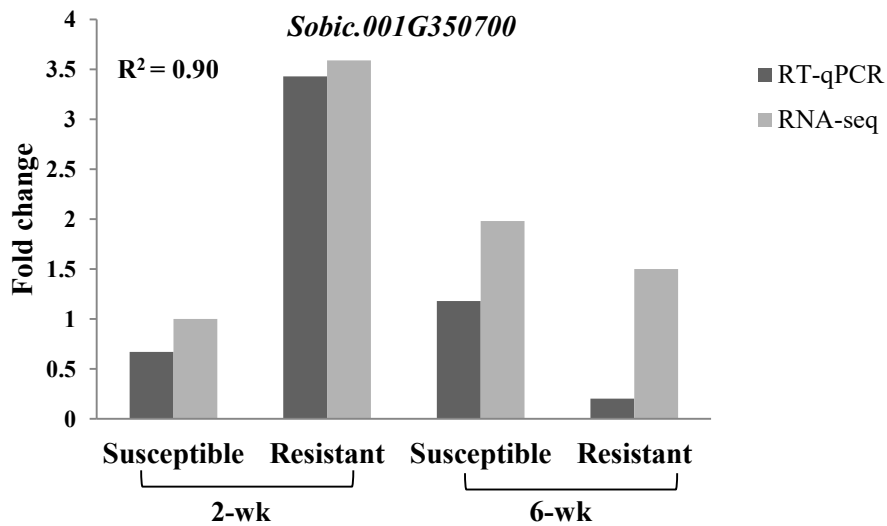

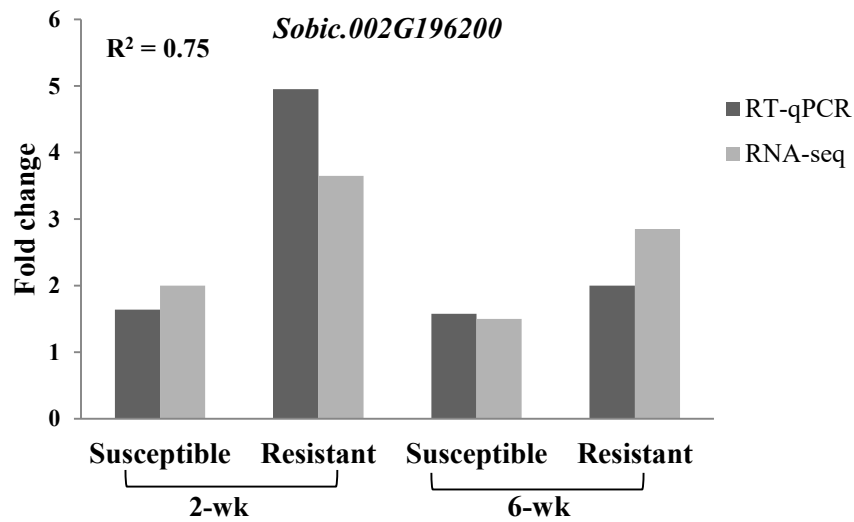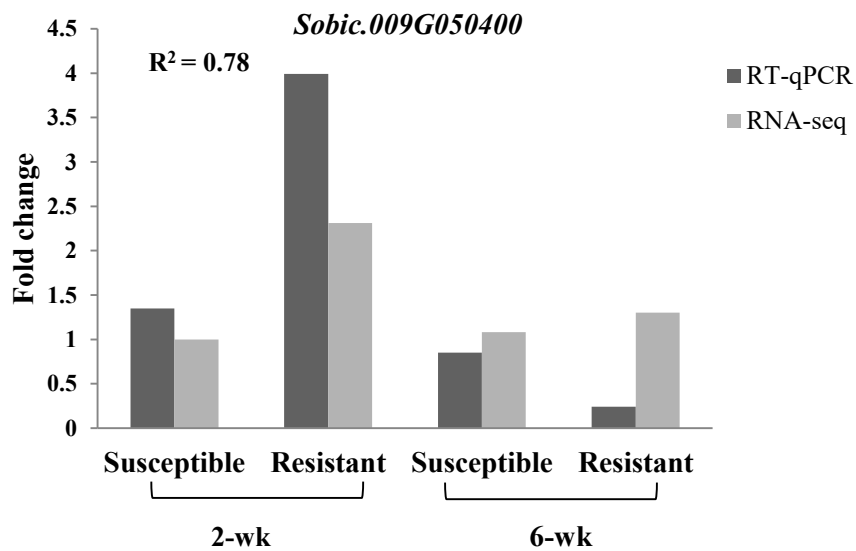

Supplement: Supplementary file 6 — Real-time quantitative RT-PCR (RT-qPCR) validation of gene expression changes detected by RNA-seq. Data are shown as a fold change of the mean expression levels from three biological replicates. The correlation between the RNA-seq data and the RT-qPCR results is shown by Pearson’s correlation coefficient (R2). (PDF 140 kb) [file 12864_2018_5095_MOESM6_ESM.pdf]
