# Supplementary material for: Effects of sugarcane aphid herbivory on transcriptional responses of resistant and susceptible sorghum
Source: BMC Genomics. 2018 Oct 26;19:774. doi: 10.1186/s12864-018-5095-x (PMC6204049; doi:10.1186/s12864-018-5095-x)
Supplement: Supplementary file 7 — Primers sequences used for RT-qPCR amplification of the five differentially expressed genes selected for validation. (DOCX 16 kb) [file 12864_2018_5095_MOESM7_ESM.docx]

**Additional file 7: Primers sequences used for RT-qPCR amplification of the five differentially expressed genes selected for validation**

| Gene ID | Primers sequences |
| --- | --- |
| *Sobic.001G143000* | F:5'-TCCGAGCTTATCTCCCTCTTCCA-3' |
|  | R:5'-ACCACATCCTCATCCAGTGCTTC-3' |
| *Sobic.001G343900* | F:5'-ACTGACAGCACCAAGATCACAGC-3' |
|  | R:5'-TCACAGGTGTAGCATCTGAAGGG-3' |
| *Sobic.001G350700* | F:5'-TTCGCCGTCAAGTTCATCGAGAG-3' |
|  | R:5'-TGCCAAATGTGTGGGAGTTAGCA-3' |
| *Sobic.002G196200* | F:5'-AACAACGCTGTGCCCAGTTCTAC-3' |
|  | R:5'-CCTCTGCTGGCTAAGAACATGAGT-3' |
| *Sobic.009G050400* | F:5'-ATGTTGCTGGGAACTCCGTTAAGT-3' |
|  | R:5'-GTGAGGCATATCTTGCGGGCTA-3' |
| *CYP* | F:5'-GTATCTGTGCTCGCCGTCTCT-3' |
|  | R:5'-TTCACCCAACTCCTCAACCCC-3' |
